# Local and Distributed Machine Learning for Inter-hospital Data Utilization: An Application for TAVI Outcome Prediction

**DOI:** 10.3389/fcvm.2021.787246

**Published:** 2021-11-12

**Authors:** Ricardo R. Lopes, Marco Mamprin, Jo M. Zelis, Pim A. L. Tonino, Martijn S. van Mourik, Marije M. Vis, Svitlana Zinger, Bas A. J. M. de Mol, Peter H. N. de With, Henk A. Marquering

**Affiliations:** ^1^Department of Biomedical Engineering and Physics, Amsterdam University Medical Centers, University of Amsterdam, Amsterdam, Netherlands; ^2^Department of Radiology and Nuclear Medicine, Amsterdam University Medical Centers, University of Amsterdam, Amsterdam, Netherlands; ^3^Department of Electrical Engineering, Eindhoven University of Technology, Eindhoven, Netherlands; ^4^Department of Cardiology, Catharina Hospital, Eindhoven, Netherlands; ^5^Heart Centre, Amsterdam University Medical Centers, University of Amsterdam, Amsterdam, Netherlands

**Keywords:** transcatheter aortic valve implantation (TAVI), outcome prediction, prognosis, mortality prediction, inter-center cross-validation, machine learning, distributed learning, aortic valve disease

## Abstract

**Background:** Machine learning models have been developed for numerous medical prognostic purposes. These models are commonly developed using data from single centers or regional registries. Including data from multiple centers improves robustness and accuracy of prognostic models. However, data sharing between multiple centers is complex, mainly because of regulations and patient privacy issues.

**Objective:** We aim to overcome data sharing impediments by using distributed ML and local learning followed by model integration. We applied these techniques to develop 1-year TAVI mortality estimation models with data from two centers without sharing any data.

**Methods:** A distributed ML technique and local learning followed by model integration was used to develop models to predict 1-year mortality after TAVI. We included two populations with 1,160 (Center A) and 631 (Center B) patients. Five traditional ML algorithms were implemented. The results were compared to models created individually on each center.

**Results:** The combined learning techniques outperformed the mono-center models. For center A, the combined local XGBoost achieved an AUC of 0.67 (compared to a mono-center AUC of 0.65) and, for center B, a distributed neural network achieved an AUC of 0.68 (compared to a mono-center AUC of 0.64).

**Conclusion:** This study shows that distributed ML and combined local models techniques, can overcome data sharing limitations and result in more accurate models for TAVI mortality estimation. We have shown improved prognostic accuracy for both centers and can also be used as an alternative to overcome the problem of limited amounts of data when creating prognostic models.

## Introduction

Transcatheter Aortic Valve Implantation (TAVI) is a consolidated procedure for aortic stenosis treatment. To support patient selection, traditional risk stratification models, either for general cardiac surgery or TAVI specific, are used for mortality estimation (1, 2). Other models, exploiting more complex algorithms, have shown higher accuracies when compared to traditional logistic regression-based models (3, 4). Nevertheless, mortality estimation models have shown limited prediction accuracy when tested on other center's populations than the one used to generate the models (5–8). This can be explained by the different distribution in the populations, given by different patient selection or practice variation among institutions.

Mitigating the models' accuracy drop on different populations is essential to obtain models with higher generalization capability. For this purpose, model updating or fine-tuning have been used successfully (9, 10). These techniques consist of making small adjustments in the model, using data from a different population, to make the models more robust for that specific population and achieve higher accuracies. It is also known that machine learning (ML) models usually benefit from a large amount of data, allowing to learn complex non-linear interactions among variables. Ideally, a single model would be developed using data from multiple centers to optimize the model's accuracy. As a practical alternative, models can be iterated by making small adjustments for each population. Sharing data between centers, however, is a complex procedure because of regulations dealing with patient's privacy and, therefore, this is not always possible in practice because of data protection regulations such as the European General Data Protection Regulation (11).

One possible approach to overcome the data sharing limitation is by exploiting distributed ML techniques. These techniques allow the training of models at multiple physical locations, regardless of their geographical distance, with limited or no data sharing. A popular distributed ML strategy, called Cyclical Weight Transfer (CWT), consists of sharing a single model across locations sequentially and cyclically for incremental updates. At each location, the model is modified using the data available at that center before sending it to the next location. This approach has been used to train deep learning models with medical images, achieving similar results as if the data was located in a single location (12). A simpler approach is to combine models trained locally at different locations. This can be achieved by using stacking ensemble, where the prediction probabilities of the models trained locally are used as features to fit a logistic regression (LR) model (13, 14). With these approaches, the models are expected to have a higher reliability and achieve better generalization capability.

In this study, we exploited two techniques to deal with the data sharing limitation to potentially improve the accuracy of models for 1-year TAVI mortality prediction. To this end, we trained multiple models based on CWT and stacking approaches across two centers without data sharing.

## Methods

### Population

Models to predict 1-year modality were created with data from a total of 1,791 patients who underwent TAVI procedures in two distinct centers were included in this study. The Amsterdam UMC—Location AMC (AMC) with 1,160 consecutive patients (first dated October 2007 and last dated April 2018) and the Catharina Hospital of Eindhoven (CZE) with 631 consecutive patients (first dated January 2015 and last dated December 2018). The 1-year mortality information was collected from a follow-up study for the AMC and by the national census for the CZE. Patients with missing outcome or with more than 50% of missing data were excluded from the study. This study, considering also where the data were located, was performed at the Amsterdam UMC and the Eindhoven University of Technology for the CZE.

### Pre-processing

Only variables that were available in both datasets were included while missing values were imputed with the mean for numerical variables and the mode for categorical variables. The measures of central tendency used for imputation were calculated for each center and used to impute it owns center's data.

Additional pre-processing was applied to the data for the development of the Neural Networks (NN) to facilitate its convergence. The continuous variables were standardized by removing their mean and by scaling them to unit variance while one-hot encoding was applied for categorical features. These steps are not required for the other classifiers.

Two approaches were evaluated to deal with the class imbalance during training: class weighting and random over-sampling. The first approach consists of assigning different weights to balance the loss of the two classes during training. The second approach consists of randomly oversampling the samples of the minority class.

### Model Development

In this study, we evaluated four distinct classifiers: Random Forest (RF), Extreme Gradient Boosting (XGB) (15), CatBoost (CATB) (16), and NN. Two NN architectures were evaluated: a narrow and a wide. The narrow is composed by two layers of 8 and 4 neurons while the wide is composed by two layers with 100 and 40 neurons. The complete architectures are described in the Supplementary Table 1. All experiments were performed on Python 3.6.9 and scikit-learn library 0.21.3 (17). We used a CWT approach to train the models with data from both centers in an iterative fashion. Besides that, we also evaluated stacking models trained individually for each center. For this, prediction outputs from models trained on each center were used to train a LR model and obtain a unique prediction output.

### Cyclical Weight Transfer Approach

**Figure 1:**
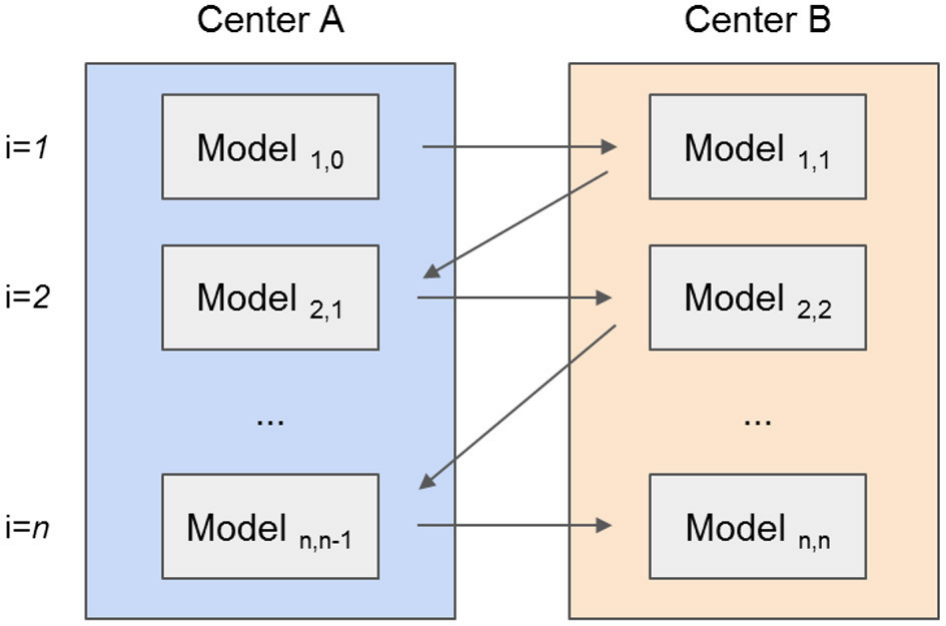
Illustration of the development of a prognostic model using the Cyclic Weight Transfer approach. The model is being trained by two different centers in an iterative fashion. Each model version is trained and exchanged between centers for *n* iterations or until a stopping criterion is reached.

The CWT approach is slightly different for the NN and the tree-based models. In CWT, as illustrated in Figure 1, the NN weights are initialized by one center and sent to the other center for updating the weights with the other center's data. This updating procedure continues until the stopping criteria is reached. Dropout was included between layers to randomly prevent some neurons from being updated by the training center (18).

**Figure 2:**
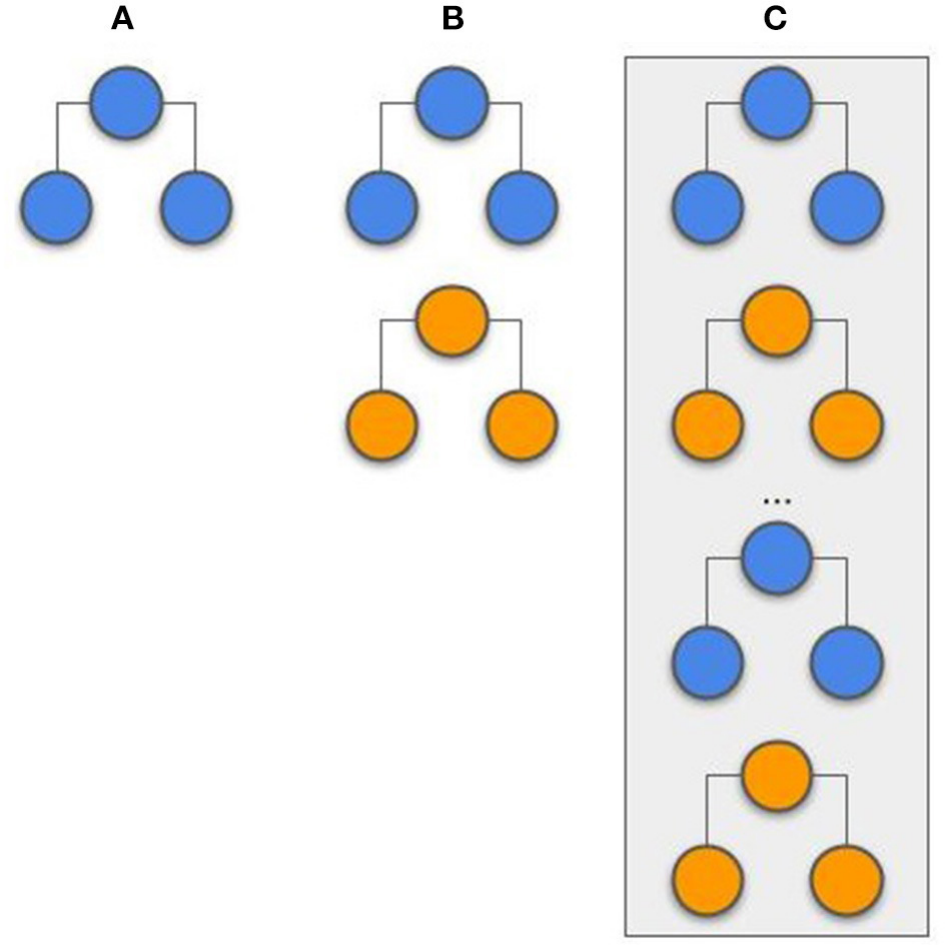
Example of a tree-based model (random forest) that is created by two different centers (represented by different colors). **(A)** A predefined number of trees is initially created by the first center. **(B)** The second center adds new trees to the forest, without modifying the previous trees. **(C)** The random forest training process is complete, with the same number of trees from each center.

The tree-based models (RF, CATB, and XGB) were trained by adding new trees, from each center, at every new iteration. To this end, the models were exchanged iteratively between centers resulting in the forest to grow. For example, as illustrated in Figure 2, an initial model created with a single tree for the first center is sent to the second center, where a new tree is added. This exchanging iterative process continues until the stopping criterion is reached: a maximum number of iterations (500) or the validation error stopped decreasing for both centers after 10 epochs. Although the trees created by one center are never modified by the other, the model is iteratively being updated by the addition of new trees from each center. For the XGB and CATB, the previous trained trees are taken in consideration when fitting new trees.

The center with the largest amount of data was used to start the training process. The hyperparameters and architectures were empirically optimized. Information regarding the values for which the hyperparameter optimization was performed can be found in the Supplementary Table 2.

### Stacking Approach

**Figure 3:**
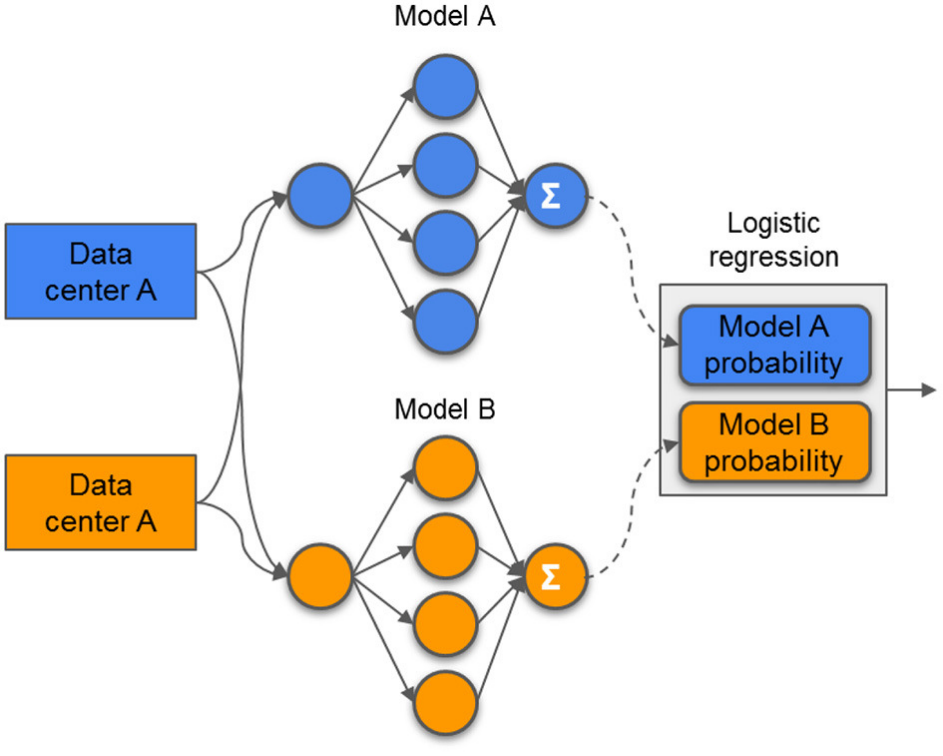
Example of a stacking model. The models are trained independently on each center and its prediction probabilities are used as features to train a single logistic regression model.

Stacking has been successfully applied in previous studies (19, 20). At the initiation of the process, the models were trained locally at each center. To this, the hyperparameters were optimized via grid search with 5-fold cross-validation. The evaluated hyperparameters are presented in the Supplementary Table 3. After both centers had their models trained, they were used to compute the probability output for all samples (training and testing). The probability output from both center's training set was used as features (2 features in total; the probability from center A and the probability from center B) to train an LR model. The probability output from the test samples was used to evaluate the LR model. With this approach, represented in Figure 3, the models and probability outputs from both centers were exchanged only once. Different classifiers were not stacked together (i.e., the NN from center A was only combined with the NN from center B).

### Internal Evaluation

To evaluate the value of creating models using data from 2 centers, we compared these models with the models that were trained on the data from only 1 center. These mono-center models were trained locally and tested on its own data. The optimization and evaluation of these models was the same as the used for the stacked approach, with hyperparameter optimization via grid search and evaluation with a 5-fold cross-validation scheme. These models have already been developed in a previous study (7).

### Evaluation

The models were evaluated with stratified 20-fold cross-validation. With this, each center split its own data in 20-folds, leading to twenty iterations with different test sets. The testing folds were kept unused until the final evaluation. The area under the curve (AUC) of the receiver operating characteristic (ROC) was used to evaluate each model. The average of the twenty AUCs, as well as the standard deviation (std), was reported for each center.

## Results

Among all 1,791 patients from two centers include on this study, 188 patients (10%) did not survive through the first year after TAVI. The baseline characteristics of the patients from both centers are summarized in Table 1.

**Table 1:** Descriptive statistics of the study group, mean ± SD or *N* (%).

**Variable**	**Instances**	**Center A**		**Center B**	
		**Survived** **(*n* = 1,039)**	**Non-survived** **(*n* = 121)**	**Survived** **(*n* = 564)**	**Non-survived** **(*n* = 67)**
Sex	Male	587 (56%)	61 (50%)	303 (54%)	46 (69%)
	Female	452 (44%)	60 (50%)	261 (46%)	21 (31%)
Age (year)		81 ± 7	83 ± 7	81 ± 6	80 ± 6
Chronic obstructive pulmonary disease	No	755 (73%)	66 (55%)	464 (83%)	48 (71%)
	Yes	282 (27%)	55 (45%)	98 (17%)	19 (28%)
Diabetes	No	720 (69%)	79 (65%)	241 (75%)	47 (70%)
	Yes	313 (30%)	42 (35%)	142 (25%)	20 (30%)
Body mass index (kg/m2)		28 ± 5	27 ± 6	27 ± 4	26 ± 4
Creatinine (μmol/L)		98 ± 41	120 ± 56	108 ± 62	116 ± 46
Smoking	No	479 (46%)	59 (49%)	392 (70%)	51 (76%)
	Former	456 (44%)	49 (40%)	41 (7%)	4 (6%)
	Yes	104 (10%)	13 (11%)	131 (23%)	12 (18%)
Beta blockers class of medicine	No	596 (57%)	80 (66%)	498 (88%)	53 (79%)
	Yes	437 (42%)	40 (33%)	66 (12%)	14 (21%)
Hemoglobin (mmol/L)		7.8 ± 1	7.7 ± 1	7.9 ± 1.0	7.5 ± 0.9
QRS complex time (msec)		104 ± 26	107 ± 27	110 ± 29	121 ± 33
Aortic valve area (cm2)		0.8 ± 0.2	0.8 ± 2	0.7 ± 0.2	0.7 ± 0.2
Aortic valve peak gradient (mmHg)		68 ± 23	64 ± 26	77 ± 24	71 ± 32
Aortic valve mean gradient (mmHg)		43 ± 16	44 ± 19	46 ± 16	39 ± 20
Previous myocardial infarction	No	851 (82%)	91 (75%)	387 (79%)	36 (69%)
	Yes	187 (18%)	30 (25%)	105 (21%)	16 (31%)
New York Heart Association (NYHA) functional classification	1	28 (3%)	1 (1%)	9 (3%)	4 (9%)
	2	220 (21%)	17 (14%)	59 (20%)	5 (12%)
	3	473 (46%)	82 (68%)	184 (61%)	24 (57%)
	4	76 (7%)	21 (17%)	49 (16%)	9 (21%)
Previous devices (such as pacemaker)	No	937 (90%)	104 (86%)	417 (89%)	36 (75%)
	Yes	102 (10%)	17 (14%)	52 (11%)	12 (25%)

**Figure 4:**
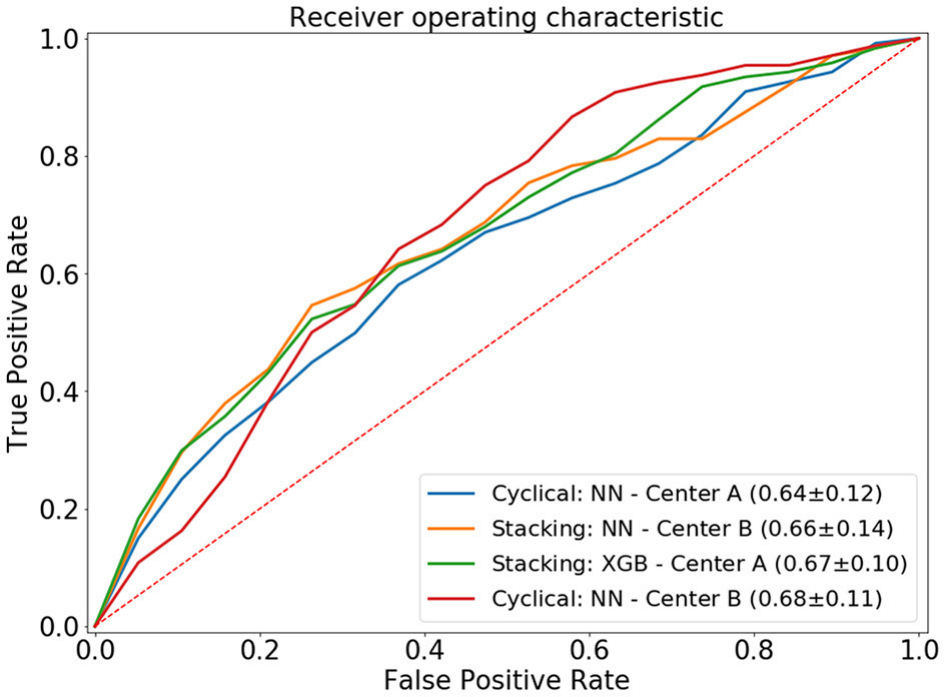
Average ROC curve (standard deviation) of the 20-fold cross-validation for the distributed and combined local models. NN, neural network; XGB, XGBoost.

The cyclical NN model with a narrow architecture achieved the highest average ROC AUC of 0.66 (center A: 0.64 AUC, center B: 0.68 AUC). This NN also achieved the highest score for center A. The stacked models with the highest accuracies achieved a ROC AUC of 0.65. This accuracy was achieved by three models; CATB (center A: 0.64 AUC, center B: 0.65 AUC), XGB (center A: 0.67 AUC, center B: 0.63 AUC) and the NN with a narrow architecture (center A: 0.64 AUC, center B: 0.65 AUC). The stacked XGBoost achieved the highest individual accuracy for center B. In Figure 4 we show the average ROC of the models with highest AUCs and in Table 2 we present all results.

**Table 2:** Average area under the receiver operating characteristic curve and its standard deviation for all experiments.

	**Model**	**Center A (*n* = 1,160)**		**Center B (*n* = 631)**		**Average of centers**	
		**Balanced class weight**	**Random oversampling**	**Balanced class weight**	**Random oversampling**	**Balanced class weight**	**Random oversampling**
Cyclical	XGBoost	0.58 ± 0.10	0.58 ± 0.10	0.62 ± 0.16	0.54 ± 0.15	0.60 ± 0.13	0.56 ± 0.13
	CatBoost	0.62 ± 0.15	0.60 ± 0.14	0.61 ± 0.14	0.61 ± 0.16	0.62 ± 0.15	0.61 ± 0.15
	Random forest	0.62 ± 0.11	0.61 ± 0.12	0.64 ± 0.13	0.64 ± 0.14	0.63 ± 0.12	0.63 ± 0.13
	Neural network (wide)	0.62 ± 0.14	0.63 ± 0.14	0.67 ± 0.14	0.65 ± 0.17	0.65 ± 0.14	0.64 ± 0.16
	Neural network (narrow)	**0.64 ± 0.12**	0.62 ± 0.13	**0.68 ± 0.12**	0.62 ± 0.15	**0.66 ± 0.12**	0.62 ± 0.14
Stacking	XGBoost	**0.67 ± 0.10**	0.61 ± 0.08	0.63 ± 0.17	0.60 ± 0.13	**0.65 ± 0.14**	0.61 ± 0.11
	CatBoost	0.64 ± 0.11	0.62 ± 0.10	0.65 ± 0.16	0.62 ± 0.13	**0.65 ± 0.14**	0.62 ± 0.12
	Random forest	0.63 ± 0.10	0.60 ± 0.09	0.64 ± 0.15	0.63 ± 0.15	0.64 ± 0.13	0.62 ± 0.12
	Neural network (wide)	0.64 ± 0.13	0.62 ± 0.13	0.64 ± 0.14	0.61 ± 0.11	0.64 ± 0.14	0.62 ± 0.12
	Neural network (narrow)	0.64 ± 0.12	0.65 ± 0.13	**0.66 ± 0.14**	0.59 ± 0.14	**0.65 ± 0.13**	0.62 ± 0.14
Mono-center	XGBoost	**0.65 ± 0.11**	0.59 ± 0.11	0.59 ± 0.17	0.56 ± 0.18	0.62 ± 0.14	0.58 ± 0.15
	CatBoost	0.63 ± 0.11	0.59 ± 0.12	0.60 ± 0.15	**0.64 ± 0.17**	0.62 ± 0.13	0.62 ± 0.15
	Random forest	0.65 ± 0.10	0.59 ± 0.11	0.62 ± 0.14	0.62 ± 0.16	**0.64 ± 0.12**	0.61 ± 0.14
	Neural network (wide)	0.64 ± 0.11	0.62 ± 0.13	0.63 ± 0.15	0.61 ± 0.15	**0.64 ± 0.13**	0.62 ± 0.14
	Neural network (narrow)	0.63 ± 0.12	0.58 ± 0.12	0.65 ± 0.16	0.60 ± 0.16	**0.64 ± 0.14**	0.59 ± 0.14

*The rows are the classifiers on different setups (cyclical, stacking or internal validation) and the columns are different balancing techniques per center. Highest accuracies per center and on average are highlighted in bold*.

The highest average accuracy for the mono-center models was a ROC AUC of 0.64, achieved by CATB, RF and the NN with narrow architecture. The highest individual accuracy was achieved by XGB for center A (AUC of 0.65) and CATB for center B (AUC of 0.64).

## Discussion

Our proposed approaches of distributed and combined local models to predict 1-year TAVI mortality with data from two centers outperformed the models trained with each center individually (mono-center). T approaches do not require the data to be sent from center to center once each center process its own data. Additionally, the centers benefited from training the models using these approaches, once their accuracies outperformed the accuracies of the mono-centers models (trained locally and independently). For both centers, the combined prediction models outperformed the models using only the local data. These approaches can be extended to multiple centers or different problems, not being exclusive for TAVI.

Some recent studies presented ML models for TAVI outcome prediction. In previous studies, Lopes et al. (3, 4) developed pipelines for outcome prediction for individual centers. Additionally, Al-Farra et al. (6) and Mamprin et al. (7) showed the accuracy drop on the evaluation of previous traditional risk scores or recent ML models when evaluated on different populations. The importance of model updating was highlighted by Lopes et al. (9) and Al-Farra et al. (10), where NN and LR models were updated after the training process was complete. They concluded that model updating is of utmost importance when using the models on different (external) populations.

This study suffered from some limitations. Some important features, which have shown prognostic value in previous studies, were not included in this study because these were not similarly reported by both centers. Also, to be aligned with previous studies, a simple imputation technique was used instead of a multiple imputation. Additionally, although center A has almost twice the number of patients from center B, the data acquisition period is relatively large (11 years, compared to 4 years from center B). This might affect the accuracy of the models since the TAVI procedures are constantly improving, from patient selection to the procedure itself, and the effects of these changes are not included in the models. Regarding the distributed experiments, the hyperparameter optimization process was reduced to a limited number of options and not many optimizations were implemented since this was not the subject of the study. Numerous additional settings could be adjusted for cyclical training: for example, the NN could be trained for multiple epochs or on mini-batches, weights could be assigned to the loss to deal with different population sizes, or even a combined loss could be taken into account when back-propagating the loss.

## Conclusion

In our study, we demonstrate two approaches to overcome the data sharing limitations between medical centers. For both centers, the combined models outperformed models in which only patients from their own center was used: for the larger center, the stacking approach showed the highest accuracy and for the smaller center, the distributed approach achieved the highest accuracy. The highest accuracy improvement was achieved for the center with a smaller number of patients, showing that when limited amounts of data are involved in creating prognostic ML models, federated can be successful option to generate a unique model in a cooperative fashion.

## Data Availability Statement

The data presented in this study are not publicly available due to privacy and ethical restrictions. Data for CZE-TU/e were obtained from Catharina Hospital (Eindhoven, the Netherlands) and have been made available for Eindhoven University of Technology (Eindhoven, the Netherlands) after the permission and approval, through formal request, of the Catharina Hospital ethical committee. Data for AMC were collected in Amsterdam UMC (Amsterdam, the Netherlands). Requests to access these datasets should be directed to RL, r.riccilopes@amsterdamumc.nl; MM, m.mamprin@tue.nl.

## Ethics Statement

The study for CZE-TU/e was approved by the Institutional Review Board (or Ethics Committee) of Catharina Hospital (Eindhoven, the Netherlands) (protocol code W18.194 with date of approval 8 November 2018). The Ethics Committee (METC) of the Amsterdam UMC (AMC) approved the research with a waiver. All data were entered into a dedicated prospective TAVI registry with an active follow-up of clinical and patient-reported outcomes. The ethics committee waived the requirement of written informed consent for participation.

## Author Contributions

RL and MM: conceptualization, methodology, software, and visualization. MM, RL, and JZ: data curation. RL: writing—original draft preparation. MM, RL, JZ, PT, SZ, MV, MM, BdM, PdW, and HM: writing—review and editing. MM, HM and PdW: funding acquisition. All authors contributed to the article and approved the submitted version.

## Funding

This work was funded by ITEA3 PARTNER (16017).

## Conflict of Interest

The authors declare that the research was conducted in the absence of any commercial or financial relationships that could be construed as a potential conflict of interest.

## Publisher's Note

All claims expressed in this article are solely those of the authors and do not necessarily represent those of their affiliated organizations, or those of the publisher, the editors and the reviewers. Any product that may be evaluated in this article, or claim that may be made by its manufacturer, is not guaranteed or endorsed by the publisher.
