# Supplementary material for: Local and Distributed Machine Learning for Inter-hospital Data Utilization: An Application for TAVI Outcome Prediction
Source: Front Cardiovasc Med. 2021 Nov 12;8:787246. doi: 10.3389/fcvm.2021.787246 (PMC8632813; doi:10.3389/fcvm.2021.787246)
Supplement: Supplementary file 1 [file Table_1.docx]

**Supplementary table S1.** Evaluated neural networks architectures.

| **Architecture** | **Layer** | **Extra param** | |  |  |
| --- | --- | --- | --- | --- | --- |
| Narrow | Dense(8) | kernel reg.=l2(0.001), activity reg.=l2(0.001) | | | |
|  | LeakyReLU(0.01) |  |  |  |  |
|  | Dropout(0.5) |  |  |  |  |
|  | Dense(4) | kernel reg.=l2(0.001), activity reg.=l2(0.001) | | | |
|  | LeakyReLU(0.01) |  |  |  |  |
|  | Dropout(0.5) |  |  |  |  |
|  | Dense(1) | kernel reg.=l2(0.001), activity reg.=l2(0.001) | | | |
|  | Sigmoid() |  |  |  |  |
| Wide | Dense(100) | kernel reg.=l2(0.001), activity reg.=l2(0.001) | | | |
|  | LeakyReLU(0.01) |  |  |  |  |
|  | Dropout(0.5) |  |  |  |  |
|  | Dense(40) | kernel reg.=l2(0.001), activity reg.=l2(0.001) | | | |
|  | LeakyReLU(0.01) |  |  |  |  |
|  | Dropout(0.5) |  |  |  |  |
|  | Dense(1) | kernel reg.=l2(0.001), activity reg.=l2(0.001) | | | |
|  | Sigmoid() |  |  |  |  |
