# Supplementary material for: Local and Distributed Machine Learning for Inter-hospital Data Utilization: An Application for TAVI Outcome Prediction
Source: Front Cardiovasc Med. 2021 Nov 12;8:787246. doi: 10.3389/fcvm.2021.787246 (PMC8632813; doi:10.3389/fcvm.2021.787246)
Supplement: Supplementary file 2 [file Table_2.docx]

**Supplementary table S2.** Hyperparameter used and searched to train the distributed models.

| **Classifier** | **Param** | **Value** |  |
| --- | --- | --- | --- |
| Tree-based | Trees | 1, 3, 5 |  |
|  | Depth | 4, 5, 6 |  |
|  |  |  |  |
| Neural networks | Learning rate | 0.01, 0.001, 0.0001 | |
|  | Optimizer | Adam |  |
|  | Min. epochs | 10 |  |
|  | Max. epochs | 500 |  |
|  | Early stopping | 10 |  |
