# Supplementary material for: Local and Distributed Machine Learning for Inter-hospital Data Utilization: An Application for TAVI Outcome Prediction
Source: Front Cardiovasc Med. 2021 Nov 12;8:787246. doi: 10.3389/fcvm.2021.787246 (PMC8632813; doi:10.3389/fcvm.2021.787246)
Supplement: Supplementary file 3 [file Table_3.docx]

**Table S3:** Hyperparameters grid used for RF, XGB, CATB, and NN.

| **Classifier** | **Parameter name** | **Parameter value** |
| --- | --- | --- |
| **RF** | Number of trees | [20, 50, 100, 200] |
|  | Max features | [auto] |
|  | Max depth | [2, 3, 4, 8] |
|  | Min samples per split | [2, 4, 6] |
|  | Min samples per leaf | [1, 2, 4] |
|  | Class weight | [Balanced] |
| **XGB** | Number of trees | [500] |
|  | Max features | [None, auto] |
|  | Max depth | [2, 3, 4] |
|  | Gamma | [0, 0.5, 1, 3] |
|  | Subsample | [0.7, 1] |
|  | Learning rate | [0.1, 0.01, 0.001] |
|  | Col sample by tree | [0.7, 1] |
|  | Scale pos weight | [1, 2, 3] |
|  | Min child weight | [1, 5, 10] |
| **CATB** | Number of trees | [500] |
|  | Max depth | [2, 3, 4] |
|  | Gamma | [0, 0.5, 1, 3] |
|  | L2 leaf reg | [3, 10] |
|  | Learning rate | [0.05, 0.10, 0.15] |
|  | Auto class weights | [Balanced] |
| **NN** | Learning rate | 0.01, 0.001, 0.0001 |
|  | Architecture | [Narrow, Wide] |
